# Early-career training in neurosurgery post-COVID: simulation, mentorship, and minimal case-volume concerns

**DOI:** 10.1097/MS9.0000000000004637

**Published:** 2026-01-21

**Authors:** Tirath Patel, Ehtisham Haider, Fizza Zaheer, Muhammad Abbas, Bhumi Daishik Patel

**Affiliations:** a Department of Neurosurgery, Trinity Medical Sciences University School of Medicine, Kingstown, Saint Vincent and the Grenadines; b Department of Neurosurgery, Punjab Medical College, Faisalabad, Pakistan; c Department of Neurosurgery, FMH College of Medicine and Dentistry, Lahore, Pakistan; d Department of Neurosurgery, Ayub Medical College, Abbottabad, Pakistan; e Department of Medical Education, Windsor University School of Medicine, Cayon, Saint Kitts and Nevis

**Keywords:** global neurosurgery, mentorship, neurosurgical training, post-COVID recovery, simulation-based education

## Abstract

Early-career neurosurgeon training has not yet fully recovered from the decrease in operative exposure that began during the early COVID era. Elective case cancellations, redeployment, and limited staff access to operating rooms reduced the chances of graduated responsibility across a variety of settings and widened existing gaps in the global workforce. Much of this was addressed by numerous programs that reinforced the application of simulation using inexpensive models and virtual environments, helping keep skills developing in the absence of practical experience. It was reported that simulator hours and trainee confidence improved, although practical surgery was needed to consolidate them. Online instruction, video-conferencing dissections, and organized mentoring also assisted learning, particularly in areas where training facilities are not evenly distributed. New interest in digital tools and early uses of artificial intelligence were indicative of a larger project to create flexible training systems. Developing a systematic roadmap that brings together case-log surveillance, simulation, video assessment, stratified mentorship, and partners in networks can help stabilize and modernize early career neurosurgical education.

*To the Editor,*

Training in the neurosurgery field at early career stages has not yet been fully restored after the disruptions that began in the early stages of COVID. Trainees at all levels were significantly cut off from case volume due to reductions in elective work, staff redeployment, and operating room access restrictions. This was observed in both high-income and emerging economies, exacerbating historical disparities in the international neurosurgical workforce^[1]^. Declining routine cranial and spinal work reduced the possibility of progressive responsibility, and in most centers, junior trainees had less time to observe or participate, even in emergency work, due to deliberately small teams to reduce exposure risk.

These limits prompted programs worldwide to increase their use of simulation. Low-cost microsurgical models enabled trainees to practice procedural steps that would otherwise have been unavailable due to reduced operative exposure. Since then, published experience indicates that simulation was a fundamental educational resource at this time and remained significant as elective work gradually re-emerged^[2]^. Programs have been reported to show improvements in simulator hours and in trainee confidence in microsurgical skills, instrument handling, and clipping strategies. Even though practical surgery is required to solidify these skills, when exposure to the operating room was sporadic, structured simulation provided a stable environment.

Online classes, broadcast dissections, and case play discussions also became commonplace. These sessions increased knowledge of complex and uncommon procedures and facilitated cross-institutional learning. In most areas, this was accompanied by a revived interest in mentorship. Assigning trainees to local mentors and readily available external advisors has been shown to impact career development, particularly in low- and middle-income environments where opportunities may not be equitable^[3]^. More exhaustive surveys of young neurosurgeons indicate that there are still barriers, namely, limited mobility, a lack of funds, and uneven access to training opportunities, which have become especially evident during the pandemic recovery period^[4]^.

Alongside these changes, there was interest in digital tools and in the initial applications of artificial intelligence, as programs considered how to assist assessment, automate feedback, and supplement anatomy teaching^[5]^. These efforts are still experimental, but they indicate that a broader effort is underway to remodel training systems to make them more flexible.

A feasible program development or revision roadmap of early career programs in the post-COVID era might include:

1. Follow operative exposure using monthly case logs to detect a shortage of standard cranial and spine procedures.

2. Have a well-organized simulation program with established goals, faculty supervision, and simple measurements like simulator hours, task time, and number of errors.

3. Include routine video-based case walk-throughs devoted to anatomy, decision points, and complication management.

4. Offer graded mentorship incorporating local mentorship with external academic or professional mentorship.

5. Promote the joint venture into regional and international education networks to eliminate inconsistent differences in exposures and distribute instructional materials.

6. Evaluate the outcomes of the review program annually, based on trainee confidence scores, the number of procedures, and examination results to improve the curriculum.

Enhancing early career routes will help stabilize neurosurgical training in the post-pandemic period and build a more resilient global workforce. We urge the incorporation of structured simulation and mentorship networks into neurosurgical training programs, particularly in resource-limited settings, to avoid long-term competency gaps.

This manuscript is made compliant with the TITAN checklist to ensure transparency in the reporting of artificial intelligence^[6]^.

## Data Availability

No new dataset was generated during the study.

## Research registration unique identifying number (UIN)

Not required because the study is a letter to the editor.
